# Visualisation of upper limb activity using spirals: A new approach to the assessment of daily prosthesis usage

**DOI:** 10.1177/0309364617706751

**Published:** 2017-06-26

**Authors:** Alix Chadwell, Laurence Kenney, Malcolm Granat, Sibylle Thies, John S Head, Adam Galpin

**Affiliations:** 1 Centre for Health Sciences Research, University of Salford, Salford, UK

**Keywords:** Activity monitoring, accelerometers, myoelectric upper Limb Prosthetics, real world usage, time series visualisation

## Abstract

**Background:**

Current outcome measures used in upper limb myoelectric prosthesis studies include clinical tests of function and self-report questionnaires on real-world prosthesis use. Research in other cohorts has questioned both the validity of self-report as an activity assessment tool and the relationship between clinical functionality and real-world upper limb activity. Previously,^1^ we reported the first results of monitoring upper limb prosthesis use. However, the data visualisation technique used was limited in scope.

**Study Design:**

Methodology development.

**Objectives:**

To introduce two new methods for the analysis and display of upper limb activity monitoring data and to demonstrate the potential value of the approach with example real-world data.

**Methods:**

Upper limb activity monitors, worn on each wrist, recorded data on two anatomically intact participants and two prosthesis users over 1 week. Participants also filled in a diary to record upper limb activity. Data visualisation was carried out using histograms, and Archimedean spirals to illustrate temporal patterns of upper limb activity.

**Results:**

Anatomically intact participants’ activity was largely bilateral in nature, interspersed with frequent bursts of unilateral activity of each arm. At times when the prosthesis was worn prosthesis users showed very little unilateral use of the prosthesis (≈20–40 min/week compared to ≈350 min/week unilateral activity on each arm for anatomically intact participants), with consistent bias towards the intact arm throughout. The Archimedean spiral plots illustrated participant-specific patterns of non-use in prosthesis users.

**Conclusion:**

The data visualisation techniques allow detailed and objective assessment of temporal patterns in the upper limb activity of prosthesis users.

**Clinical relevance:**

Activity monitoring offers an objective method for the assessment of upper limb prosthesis users’ (PUs) activity outside of the clinic. By plotting data using Archimedean spirals, it is possible to visualise, in detail, the temporal patterns of upper limb activity. Further work is needed to explore the relationship between traditional functional outcome measures and real-world prosthesis activity.

## Background

Upper limb myoelectric prostheses are designed to replace the anatomical arm and restore a level of functionality in people with partial limb loss/absence. To date, clinical studies evaluating myoelectric prostheses have been limited to assessing the ability of the user to perform tasks under controlled conditions. The assessment tools used in these studies have well-known limitations^2^ and, at best, provide a ‘snapshot’ of performance on a small set of tasks, on a given day, typically under ‘ideal’ conditions. In recent years, research in the field of stroke rehabilitation has questioned the assumption that upper limb capacity, as measured using one-off clinical assessment tools, relates to upper limb usage outside of the clinic^3^ and that improvements in clinical functionality translate to real-world improvements in upper limb usage.^4,5^ These studies raise serious questions with regard to the way in which upper limb prostheses are currently evaluated.

The use or otherwise of upper limb prostheses is currently determined through self-report questionnaires, which rely on accurate and unbiased recall and provide information only on average characteristics.^6–8^ For example, the Trinity Amputation and Prosthesis Experience Scales (TAPES)^9^ asks participants ‘On average how many hours a day do you wear your prosthesis’. It is also clear that the terminology in the literature used to characterise device use and/or abandonment is inconsistent and often ill-defined, making comparisons between studies difficult. For example, the continuum between active frequent users of a prosthesis and total rejecters encompasses a range of terms including ‘active users’,^6^ ‘passive wearers … who do not use the active capabilities of their device’,^10^ ‘partially active users’,^6^ ‘occasional users’^11^ and ‘primary and secondary prosthesis rejecters’.^12^ The importance of being able to properly understand real-world use of a prosthesis is emphasised by reports of high rates of myoelectric prosthesis rejection^10^ and overuse injuries of joints and muscles.^13^

Activity monitors offer the potential to objectively characterise upper limb activity outside of the clinic. These monitors typically comprise tri-axial accelerometers, a battery, signal processing and data storage and are worn on the wrist(s). Activity monitoring has been used successfully in numerous studies to characterise upper limb usage for people recovering from a stroke.^3,14–17^ Despite the clear benefit of obtaining objective data on upper limb motion outside of the clinical environment, the authors have identified only two previous papers relating to the use of activity monitors for the assessment of people with upper limb absence.^18^ In our earlier paper,^1^ activity monitoring data were presented from two congenital prosthesis users (PUs), one reporting to be a satisfied and one a dissatisfied user of a myoelectric prosthesis. Participants were asked to wear the monitors on both wrists (anatomical and prosthesis) and, to allow comparison with previously published data, the activity monitoring data were analysed using the methods of Bailey et al.^3^

In Bailey’s study, activity monitors (Actigraph GT3X+) were worn on both wrists (monitors were only removed during bathing/showering). Using proprietary algorithms within the Actilife6 software, the data were filtered, grouped into 1-s epochs and converted into activity counts.^19^ For each second, activity counts across the three axes were summed to generate a vector magnitude $\left(\mathrm{VM}=\sqrt{x^{2}+y^{2}+z^{2}}\right)$. Bailey used these vector magnitudes to derive two variables, a bilateral magnitude (BM) representing the overall intensity of activity per second across the upper limbs $\left(\mathrm{BM}={\mathrm{VM}}_{\mathrm{Paretic}}+{\mathrm{VM}}_{\mathrm{Non}-\mathrm{paretic}}\right)$ and a magnitude ratio (MR) representing the relative contribution of each arm to the activity $\left[\mathrm{MR}={\ln(\mathrm{VM}}_{\mathrm{Paretic}}{/\mathrm{VM}}_{\mathrm{Non}-\mathrm{paretic}})\right]$. Bailey’s methods meant that unilateral activity, where the vector magnitude on one of the arms was equal to 0, generated a non-finite MR; consequently, arbitrary values were introduced for unilateral activity (MR = 7 and −7 for unilateral use of the paretic and non-paretic arms, respectively). The data were represented visually by plotting a scatter of the MR (x-axis) versus BM (y-axis) with a colour map used to represent the number of occurrences (seconds) of each point; furthermore, the median MR and BM were reported to provide summary measures of symmetricity and intensity of use.

Bailey’s methods provided a good initial insight into the upper limb data; however, the somewhat abstracted approach to presentation of the data made interpretation difficult. A simple summary of the amount of activity across the upper limbs over the monitoring period can be displayed using histograms. The measure of contribution to activity used in Bailey’s study (MR) is based off a natural log and is therefore not intuitive; additionally, due to the arbitrary value introduced for unilateral activity, the scale of MR is not continuous. In this article, we therefore propose assessing the relative contribution of each arm to the activity as a percentage. In addition, Bailey’s methods do not consider temporal patterns in prosthesis usage throughout the day. Temporal patterns may be of particular relevance in this context as users have previously reported problems of discomfort^7,12,20–22^ and battery life,^7,22–24^ both of which may lead to an increased likelihood of non-wear and/or non-use later in the day.

Visualisation of time series whole body activity data has been addressed in a previous study by Loudon and Granat.^25^ In this approach, the authors collected data from a thigh worn activity monitor (activPAL3) over a 7-day period. Data were sampled at 20 Hz and proprietary algorithms were used to allocate event markers (upright, lying or sitting) to each sample. Different visualisation methods were used to display the data, including an Archimedean spiral plot, first introduced by Carlis and Konstan.^26^ This approach is of particular interest as patterns in activity over time/between days are clearly visible. The properties of an Archimedean spiral are such that a straight line drawn from the origin will intersect each ring of the spiral at the same time point in the data.

In this article, we propose the use of simple histograms of activity counts, together with Archimedean plots to visualise upper limb activity data. The new approaches will be illustrated with example data from anatomically intact (AI) subjects and PUs. In brief, we first show how histograms of activity counts, together with simple descriptive statistics, may be used to illustrate the distribution of activity between limbs over the monitoring period. Second, we show how spiral plots offer the potential to visualise in detail the use of the participant’s upper limbs over time. Through the use of graduated colour, there is a potential to quickly see the relative dependence on a particular arm. Finally, we propose that by adapting the spiral plot it would be possible to overlay relevant events such as non-wear or hand activations to further understand patterns in usage throughout the day. In this article, we illustrate the approach by overlaying data from a wear diary onto the spiral plots.

## Methods

### Participants

Four participants were recruited: two healthy AI participants (female, age: 27 and 28 years, one left and one right-handed) recruited from the University of Salford, and two myoelectric PUs with congenital trans-radial limb loss (male, age: 44 and 45 years, one with left and one with right limb absence) recruited from the University of Salford Prosthetics and Orthotics Professional Patient Database. Both PUs were prescribed with single degree of freedom myoelectric hands. PU 1 who reported to be satisfied with his prosthesis had 1.5 years of experience with a myoelectric prosthesis; his prosthesis included a wrist rotator. PU 2 had 35 years of experience with myoelectric prostheses; he reported to be dissatisfied with his prosthesis. All participants were recruited as part of a larger pilot study for which activity monitoring was a key outcome measure.^1^ Ethical approval for the study was granted by the University of Salford School of Health Sciences Research Ethics committee (REF: HSCR 15-130) and informed consent was gained from all participants.

### Equipment

Each participant was provided with two Actigraph GT3X+ activity monitors which provide continuous logging of acceleration across three axes at 30 Hz. For PUs, one monitor was worn on the wrist of the anatomical arm and the other on the wrist of the myoelectric prosthesis; for AI participants, one monitor was worn on each wrist. Both monitors were placed on elasticated wristbands labelled as to which wrist they should be worn on and in what orientation they should be worn.

### Protocol

Participants were asked to wear the monitors for a 7-day period, only removing them when bathing. For the PUs, the monitor worn on the wrist of the myoelectric prosthesis was to remain on the myoelectric prosthesis throughout the week and not be swapped onto other prostheses the person may use. Participants were asked not to alter their behaviour during the data collection period. Each participant was also supplied with a wear diary to assist interpretation of the activity monitoring data, in which they were asked to record times when they were asleep, and when they removed the monitors or the prosthesis.

Data were downloaded, filtered (employing the low-frequency extension filter^27^) and collated into 1-min epochs (for ease of visualisation) using proprietary Actilife5 software. Furthermore, the processed data were converted into activity counts^19^ which were summed across the axes generating vector magnitudes $\left(\mathrm{VM}=\sqrt{x^{2}+y^{2}+z^{2}}\right)$. The precise algorithm for calculating counts from accelerometer signals is not provided by the manufacturer, but accelerometer counts reflect change in accelerometer readings. Hence, no movement would correspond to zero counts. The raw acceleration data, which included both true acceleration and gravity components, were also exported. Data were transferred into MATLAB (v. 2016a) for further analysis.

### Data analysis

#### Histograms

By displaying the data in the form of a histogram, it is possible to visualise the contribution of each arm to all activities undertaken throughout the recording period. The ratio of contribution to activity between the upper limbs is provided as a percentage. The percentage contribution of each arm for each epoch (minute of use) was calculated by dividing the vector magnitude on the dominant/anatomical arm by the total vector magnitude across both arms ${[\mathrm{round}(\mathrm{VM}}_{\mathrm{Dom}}{/(\mathrm{VM}}_{\mathrm{Dom}}{+\mathrm{VM}}_{\mathrm{Non}-\mathrm{Dom}})\times 100)]$; any time points where the vector magnitude across both arms was equal to 0 (no activity) were removed from the dataset. For each percentage band (0–100% in 1% increments), the time in minutes was summed; for ease of visualisation, the time was displayed on a log_10_ scale to mitigate for large amounts of unilateral activity.

#### Spiral plots

A script was written using MATLAB (The Mathworks, Inc.) to produce spiral plots from the CSV data tables exported from the Actilife software. Each epoch was marked with an event marker. Where no activity counts were recorded on either monitor (VM = 0), the epoch was marked as ‘both arms at rest’, where activity counts were only recorded on one of the arms the epoch was marked as ‘unilateral’ use of the corresponding limb, and where counts were recorded on both arms, a percentage contribution was calculated (see Histograms) and the data were split into 10% bands (Figure 2). Colours were allocated to each of these bands, complementary colours were chosen to ensure that patterns of usage would be clearly visible. The periods when there was activity recorded on either/both upper limbs, were given a gradient of colour between unilateral use of the prosthesis and unilateral use of the anatomically intact arm. A spiral was plotted in the form of a 24-h clock with midnight at the top. Data were built up day by day working out from the centre.

## Results

### Raw data

Monitors and completed wear diaries were returned by all four participants. The raw accelerations along all three axes were visually inspected and there were no cases of missing data. Comparison of the raw data with the wear diary showed some disagreement. For one PU (user 2), accelerations were recorded on the monitor worn on the wrist of the prosthesis on days when it was reported in the diary not to be worn by the user; this transpired to be due to the prosthesis being carried. Furthermore, for the same user, self-report showed the prosthesis to be worn for a full 12 h when no accelerations were recorded on the monitor. It was assumed that the user had incorrectly used the 24-h clock, reporting to don the prosthesis at 05:30 when he should have put 17:30.

### Histograms

**Figure 1.**
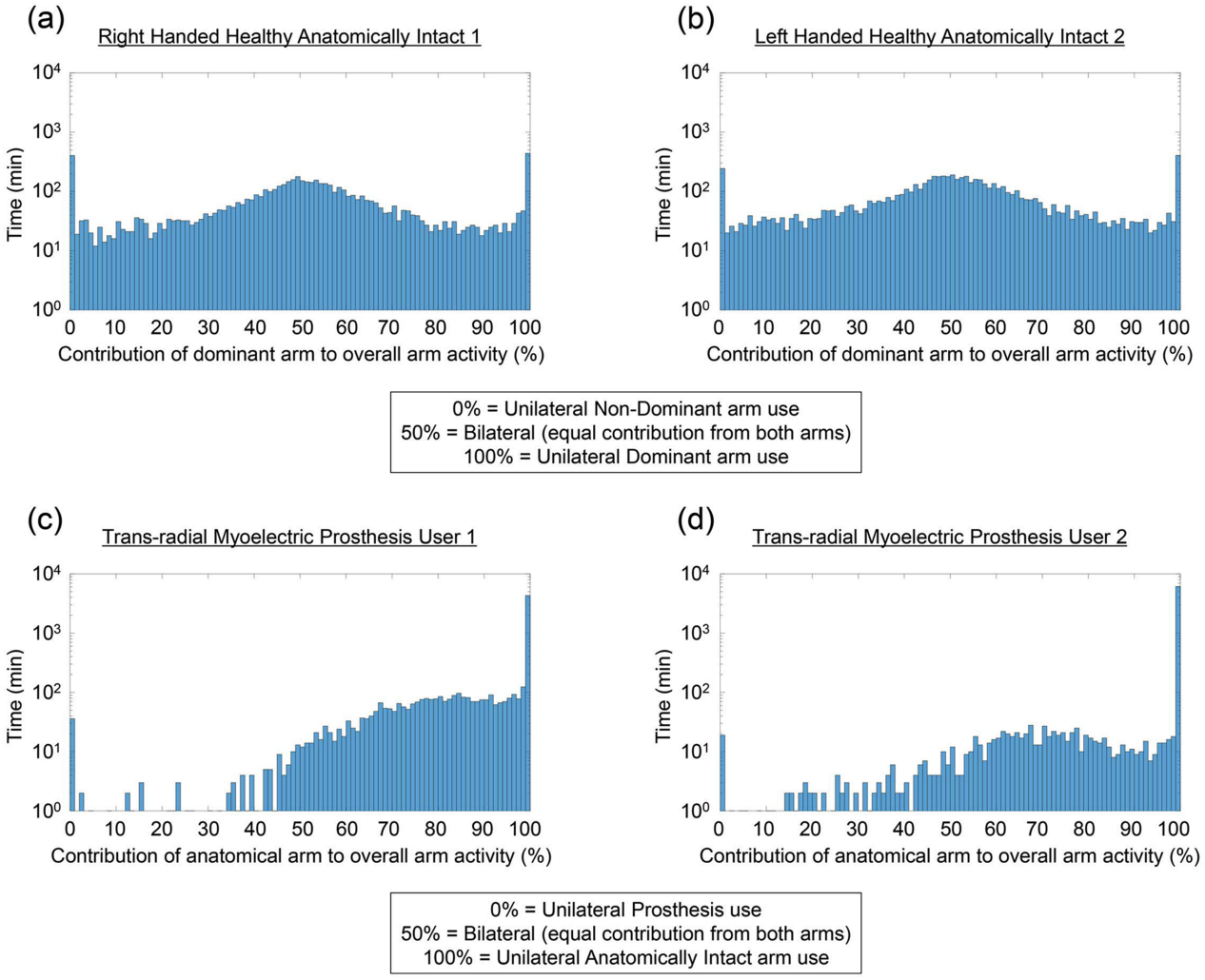
Histograms representing the balance of activity across the upper limbs. (a, b) Data recorded for the two anatomically intact participants (1 and 2, respectively) and (c, d) data for the two prosthesis users. On the x-axis, the ratio of contribution to activity between the upper limbs is shown as a percentage. 100% indicates unilateral use of the dominant/anatomical limb, 50% indicates bilateral use of the arms and 0% indicates unilateral use of the non-dominant arm/prosthesis. The data have been grouped into 1% bins, and the y-axis shows the total time in minutes, plotted using a log_10_ scale. A log_10_ scale is used for ease of visualisation of the prosthesis user data considering the large amount of unilateral activity on the anatomical arm.

For the two AI participants (Figure 1(a) and (b)), the peak in the data is centred around 50% usage of each arm. The median contribution of the dominant arm to the overall activity was 51.20% and 51.27% for participants 1 and 2, respectively. The activity for the two PUs (Figure 1(c) and (d)), however was, as expected, heavily skewed towards the anatomical arm. For both PUs, the median percentage contribution of the anatomical arm to overall upper limb activity was 100% (unilateral use of the anatomical arm). This value is biased by times when the prosthesis was removed which would also show as unilateral use of the anatomical arm; therefore, the median was re-calculated only for the times the prosthesis was worn (based on self-report). The median values were 87.64% (wear time: 69.37 h) and 87.06% (wear time: 22.05 h) for user 1 and user 2, respectively.

**Figure 2.**
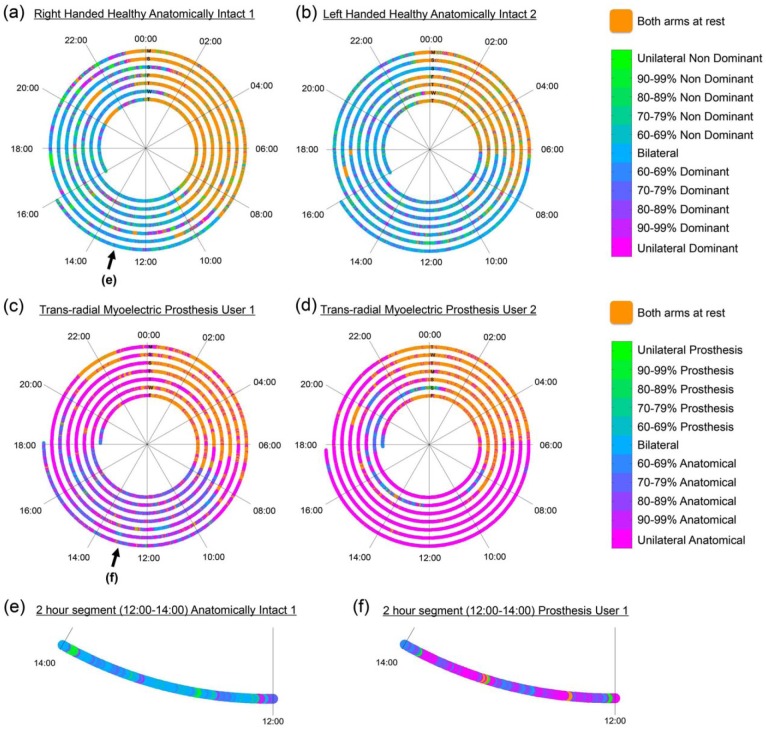
Upper limb activity recorded from two wrist worn activity monitors. Each graph (a–d) represents data recorded over a 7-day period, with each ring representing 24 h. Progression of time is from the centre outwards. Each ring is labelled with a letter signifying the day of the week corresponding to the subsequent 24 h of data. The scale in the legend displays colours relating to the ratio of activity counts recorded on each monitor. (a) Right-handed healthy anatomically intact participant, (b) left-handed healthy anatomically intact participant, (c) myoelectric prosthesis user with congenital trans-radial limb absence on the right-hand side – Prosthesis User 1 (self-reports to be satisfied with prosthesis), (d) myoelectric prosthesis user with congenital trans-radial limb absence on the left-hand side – Prosthesis User 2 (self-reports to be dissatisfied with prosthesis). (e, f) Expanded views of the 2-h segment between 12:00 and 14:00 on the final day (Monday) for (e) anatomically intact participant 1 and (f) Prosthesis User 1.

All participants demonstrate stacks of unilateral activity (0% = unilateral activity on the non-dominant/prosthesis side, 100% = unilateral activity on the dominant/anatomically intact side). The ratio of unilateral activity between the two arms (dominant ÷non-dominant or anatomical ÷ prosthesis) allows clear differentiation between healthy AI participants and PUs. Both AI participants demonstrated almost equal unilateral activity on each arm (ratio = 1.03:1 and 1.68:1). The ratio for the PUs was once again skewed by the prosthesis non-wear times (ratio = 115.42:1 and 230.21:1); however, when only the wear time was considered, both users demonstrated similar ratios to each other (ratio = 29.61:1 and 24.11:1).

### Spiral plots

In Figure 2, data are presented from the two AI participants and two PUs. Immediately, a colour difference can be seen between the two pairs of participants due to the reliance on the anatomical hand for the PUs. Furthermore, the period when the participants were asleep can also be clearly seen. Figure 2(e) and (f) shows magnified sections for AI participant 1 and PU 1 (during a period when the prosthesis was worn) highlighting differences in upper limb usage between the two. The upper limb activity for the AI participant (Figure 2(e)) is predominantly bilateral (blue), interspersed with bursts of unilateral activity on both the dominant and non-dominant sides. In comparison, the PU (Figure 2(f)) demonstrates very little unilateral prosthesis use (green) and large amounts of unilateral use of the anatomical arm (magenta); on occasions where the PU is performing bilaterally, there is a preference towards the anatomical arm as demonstrated by the purple colouring.

To demonstrate the capacity of these plots for inclusion of additional data, Figure 3 shows data from PU 1 in which self-reported removal of the prosthesis (black) has been included. If the self-report is accurate, it would be expected that during the black periods all data points would be orange or magenta (no activity on the prosthesis).

**Figure 3.**
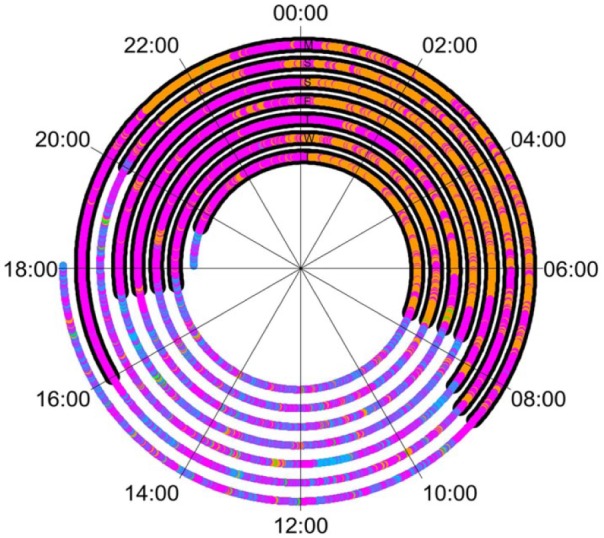
Data for Prosthesis User 1 with an underlay of information from the wear diary. The black markers represent times when the user reported removing the prosthesis, approximately from 18:00 to 08:00. It would be expected that these would align with times when only the anatomical arm showed to be active or when there was no activity on either arm.

## Discussion

Research in the field of stroke rehabilitation has highlighted both the limitations with self-report as a tool for assessment of real-world upper limb use and, perhaps more importantly, that clinically assessed upper limb functionality correlates weakly with real-world arm usage. These findings raise questions with regard to current upper limb prosthetics research. Previously, we reported the first real-world data on upper limb prosthesis use,^1^ but the data visualisation tools used were limited in scope; they did not provide a clear summary of upper limb activity over the recording period, nor did they illustrate the temporal patterns in the data.

Archimedean spirals, combined with histograms, offer a promising approach for the display of upper limb activity. Using these plots, we observed very clear differences in upper limb usage behaviours between PUs and AI participants, as well as patterns in behaviour in relation to time-of-day. The benefit of these plots over the methods used previously is that changes in the patterns of behaviour can be easily identified. For example, PU 1 (Figure 2(c)) reported to be fairly satisfied with his prosthesis; however, it is clear that he regularly removed his prosthesis around 17:00–19:00 for the remainder of the evening, shown by the large portion of magenta representing unilateral activity of the anatomical arm during the evening period (validated by comparison with the wear diary). The spiral plots also offer the potential for the display of additional data by under-laying thicker lines around the existing spiral. In future, if further data could be logged regarding the way the prosthesis is used, such as hand activations, then this could be plotted over the activity monitor data to help explain potential patterns in prosthesis use/non-use or highlight passive prosthesis use.

The use of accelerometers to characterise upper limb activity is not without its limitations.^28^ For example, the analysis of wrist-worn accelerometer data presented here does not discriminate between the swinging of the arm during walking and active functional use of the upper limb. Furthermore, the choice of epoch length can impact on the amount of unilateral activity recorded. Nevertheless, wrist-worn accelerometry has gained acceptance as an objective measure of upper limb activity outside of the clinic.^29^

The data displayed in this article are part of a larger study designed to improve our understanding of factors contributing to user performance with upper limb myoelectric prostheses. The PUs involved in the study, therefore, only wore the activity monitors on their myoelectric prostheses, despite one of them wearing an alternative prosthesis during some of the days of testing. It is therefore not possible to differentiate unilateral use of the anatomical arm from bilateral activity with either the residual limb or a secondary prosthesis. In future, studies addressing the more general question about upper limb activity should place activity monitors on the wrist of all prostheses the participant may wear (e.g. a cosmetic, or body-powered secondary prostheses). Furthermore, it may also be useful to assess the usage of the residual limb; by placing monitors on the upper part of both arms, it may be possible to gain an insight into times when the prosthesis was removed, times it may have been carried, and information about bilateral activity at times when no prosthesis was worn. These approaches, however, do raise significant questions about practicality, and until such time as prosthesis non-wear can be accurately identified, data should be considered in parallel to a wear diary. A further limitation raised in our previous paper^1^ regarded the lack of ability of the activity monitors to inform on active prosthesis use; from this data, we can only determine that there was movement of the upper limb, we cannot infer that the user was opening or closing the hand. These limitations should be considered during the analysis of the data we have presented, however, as has been highlighted in this article, there is a capacity within the spiral plot design to reflect more advanced information if it were to be available.

Finally, we have designed the spirals to display data derived from pre-processed activity counts, generated by the Actilife software. In future, it would be more beneficial to derive the percentage contribution of each arm from the raw accelerations, this would enable compatibility with activity monitors from different manufacturers.
